# Spillover of Swine Coronaviruses, United States

**DOI:** 10.3201/eid2407.172077

**Published:** 2018-07

**Authors:** Sarah N. Bevins, Mark Lutman, Kerri Pedersen, Nicole Barrett, Tom Gidlewski, Tom J. Deliberto, Alan B. Franklin

**Affiliations:** US Department of Agriculture Animal and Plant Health Inspection Service—Wildlife Services National Wildlife Research Center, Fort Collins, Colorado, USA (S.N. Bevins, M. Lutman, K. Pedersen, N. Barrett, T. Gidlewski, T.J. Deliberto, A.B. Franklin);

**Keywords:** PEDV, porcine epidemic virus, spillover, pathogen emergence, swine coronavirus, swine, pigs, viruses, United States

## Abstract

Porcine epidemic diarrhea virus, a pathogen first detected in US domestic swine in 2013, has rapidly spilled over into feral swine populations. A better understanding of the factors associated with pathogen emergence is needed to better manage, and ultimately prevent, future spillover events from domestic to nondomestic animals.

Pathogen spillover mechanisms vary, but one route involves pathogens moving from heavily infected domestic animal hosts to nondomestic hosts (*1*). These spillover and emergence events create a dynamic landscape for pathogen transmission.

Porcine epidemic virus (PEDV) is an emergent pathogen in the United States. It can cause 90%–95% mortality in young, naive pigs and substantial weight loss and dehydration in adult swine. The virus was first documented in the United States in April 2013 and spread rapidly, leading to loss of 10% of the US commercial swine population in 31 states within 18 months (*2*), which cost the industry >US $400 million. Horizontal transmission of the virus on shared agricultural resources (*3*) most likely aided its rapid spread among facilities, demonstrating the difficulty of slowing the spread of robust pathogens.

During October 2012–September 2015, we collected serum from feral swine and analyzed it for PEDV exposure. The United States has ≈5–6 million feral swine, and their populations are expanding rapidly (*4*). Although opportunities for direct contact between feral swine and pigs in biosecure swine operations are limited, interactions have been documented with smaller backyard operations, and a recent multistate brucellosis outbreak was linked to backyard pigs infected by feral swine (*5*). Disease spillover into nondomestic hosts can serve as a continuous source for reintroduction into domestic animals, complicating international trade (*6*).

Of the 7,997 feral swine samples tested (Figure), 253 tested positive by PEDV ELISA (seroprevalence 3.2% [95% CI 2.8%–3.5%]). Those 253 samples underwent additional screening, and 8 (seroprevalence 0.1% [95% CI 0.03%–0.16%]) were confirmed to be PEDV antibody positive (Technical Appendix Tables 1, 2). Two additional samples were considered suspected positives. The remaining 245 positive samples (seroprevalence 3.1% [95% CI 2.7%–3.4%]) probably represent exposure to transmissible gastroenteritis virus (TGEV) rather than PEDV (Technical Appendix Table 1).

**Figure:**
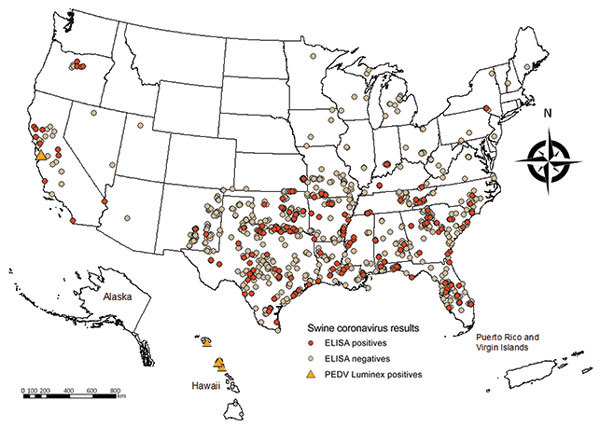
Collection locations of feral swine samples tested for exposure to swine coronaviruses, United States. In California, 4 PEDV-positive samples were detected at the same location. Samples that were ELISA-positive, but PEDV-negative probably indicate exposure to transmissible gastroenteritis virus.

The 8 PEDV-seropositive feral swine samples were from Hawaii and California (Figure). PEDV was first confirmed in California domestic swine in December 2013. The 4 positive feral swine samples from California were collected in September 2014 from adult animals in Santa Clara County. In Hawaii, seropositive feral swine were detected on Oahu and Kauai (Figure). Hawaii confirmed its first case of PEDV in domestic swine on Oahu in November 2014, but our findings identified a PEDV-positive feral swine sample collected in April 2014, before detection in domestic swine on the same island. This finding suggests initial PEDV introduction into domestic pigs in Hawaii might have gone undetected for 7 months before the first confirmed case. The 4 PEDV-positive feral swine samples from Hawaii were collected at 4 different times.

Results indicate that this newly introduced virus spilled over from domestic livestock to a nondomestic species during a relatively short period (<1 year). Prior research suggests directionality (*7*,*8*), with the virus moving from domestic swine to feral swine, rather than the reverse. Data presented here support this finding because positive feral swine were not detected until a year after detection in US domestic swine.

Biosecurity in the US commercial swine industry is comprehensive; however, the spread of PEDV demonstrates that a modern and precisely managed livestock industry is still susceptible to emergent pathogens. PEDV is relatively hardy, persisting on fomites for up to 20 days at low (4°C) temperatures (*9*). Biosecurity designed to prevent transmission of labile pathogens or to prevent introduction of a new pathogen through traditional routes may be insufficient for nonlabile pathogens introduced through new mechanisms.

The transmission pathway from infected facilities to feral swine is unknown. Previous research has detected PEDV in the environment (*3*,*7*) but did not differentiate viral RNA from infectious virus. Swine facilities often move waste to holding ponds, and these ponds could be a source of infectious virus. Infected swine in backyard operations also could facilitate spillover.

Our data also demonstrate that 3.1% of feral swine had been exposed to another coronavirus, probably TGEV. TGEV, like PEDV, is found only in swine, can survive on fomites, and can cause high mortality rates in pigs <2 weeks of age. TGEV has been found in the US domestic swine industry since the 1940s. We found TGEV-positive feral swine throughout the entire sampling period and throughout the United States (Figure; Technical Appendix Tables 1, 2), suggesting that TGEV is probably being persistently transmitted among feral swine, although continual spillover from domestic swine cannot be ruled out. Whether PEDV will display a similar pattern of endemicity over time is unknown; however, our data did not suggest continual transmission or high seroprevalence. For example, the most recent PEDV-seropositive feral swine in Hawaii was detected in January 2015. Seventy-six feral swine sampled from the same island after that date were seronegative, suggesting that either seroprevalence was low enough to evade detection or that viral transmission burned out, most likely after initial deaths of susceptible piglets. Research in Asia, however, has found higher PEDV exposure in wild boar, reinforcing that animals can survive infection and raising the possibility of continual transmission in nondomestic swine populations (*6*).

Technical Appendix Methods for study of spillover of swine coronaviruses, United States.
