# Supplementary material for: Spillover of Swine Coronaviruses, United States
Source: Emerg Infect Dis. 2018 Jul;24(7):1390–2. doi: 10.3201/eid2407.172077 (PMC6038755; doi:10.3201/eid2407.172077)
Supplement: Technical Appendix — Methods for study of spillover of swine coronaviruses, United States. [file 17-2077-Techapp-s1.pdf]

Article DOI: https://doi.org/10.3201/eid2407.172077

Spillover of Swine Coronaviruses, United States

Technical Appendix

Methods

Serum samples from 7,997 feral swine removed as part of permitted wildlife damage management activities were collected across the United States (Technical Appendix Table 1) during October 2012-September 2015. This covers both the time period before porcine epidemic virus (PEDV) was first detected in US domestic swine and the initial rapid spread of the virus in domestic swine facilities. Samples were collected throughout the entirety of the range of breeding feral swine populations and in both domestic swine PEDV-positive and PEDV-negative states.

Feral swine samples were screened using a whole-virus PEDV ELISA (*1*,*2*). The ELISA is based on a US PEDV isolate (USA/NC35140/2013) which detects IgA and IgG to PEDV strains (protoype and S INDEL) circulating in the United States. Gimenez-Lirola et al. demonstrated that cross-reactivity can occur with the PEDV ELISA against transmissible gastroenteritis virus (TGEV), but not against other swine coronaviruses, such as porcine respiratory cornonavirus, and porcine deltacoronavirus. Because of this potential cross-reactivity, all positive ELISA samples (sensitivity 88.8%, specificity 100%) were confirmed using a PEDV specific S1 multiplex fluorescent microbead-based immunoassay (*3*). Confidence intervals for seroprevalence values were calculated using the Copper-Pearson exact method. Results are broken down by both state and calendar of sample collection (Technical Appendix Tables 1, 2).

References

1. Bjustrom-Kraft J, Woodard K, Giménez-Lirola L, Rotolo M, Wang C, Sun Y, et al. Porcine epidemic diarrhea virus (PEDV) detection and antibody response in commercial growing pigs. BMC Vet Res. 2016;12:99. PubMed http://dx.doi.org/10.1186/s12917-016-0725-5

2. Chen Q, Thomas JT, Giménez-Lirola LG, Hardham JM, Gao Q, Gerber PF, et al. Evaluation of serological cross-reactivity and cross-neutralization between the United States porcine epidemic diarrhea virus prototype and S-INDEL-variant strains. BMC Vet Res. 2016;12:70. PubMed http://dx.doi.org/10.1186/s12917-016-0697-5

3. Gimenez-Lirola, LG, Zhang J, Carrillo-Avila JA, Chen Q, Magtoto R, Poonsuk K et al. (2017). Reactivity of anti-PEDV structural protein antibodies to porcine enteric coronaviruses: diagnostic implications. J Clin Microbiol. 2017;55:1426–36.

Technical Appendix Table 1. Feral swine sample sizes by state, United States*

State/ Territory	No.	No. coronavirus-positive	Coronavirus seroprevalence (95% CI)	No. PEDV-positive	PEDV seroprevalence (95% CL)
Alabama	283	10	0.04 (0.02–0.06)	0	0
Arkansas	476	14	0.03 (0.02–0.05)	0	0
Arizona	67	1	0.01 (0–0.08)	0	0
California	641	18	0.03 (0.02–0.04)	4	0.006 (0–0.02)
Colorado	5	0	0 (0–0.52)	0	0
Florida	843	0	0 (0–0.01)	0	0
Georgia	440	12	0.03 (0.01–0.05)	0	0
Guam	12	0	0 (0–0.26)	0	0
Hawaii	443	18	0.04 (0.02–0.06)	4	0.009 (0–0.02)
Iowa	4	0	0 (0–0.60)	0	0
Illinois	36	0	0 (0–0.10)	0	0
Indiana	83	3	0.04 (0.01–0.10)	0	0
Kansas	251	11	0.04 (0.02–0.08)	0	0
Kentucky	25	1	0.04 (0–0.20)	0	0
Louisiana	398	14	0.04 (0.02–0.06)	0	0
Maine	2	0	0 (0–0.84)	0	0
Michigan	31	0	0 (0–0.11)	0	0
Minnesota	1	0	0 (0–0.98)	0	0
Missouri	171	4	0.02 (0.01–0.06)	0	0
Mississippi	389	15	0.04 (0.02–0.06)	0	0
North Carolina	320	5	0.02 (0.01–0.04)	0	0
New Hampshire	5	0	0 (0–0.52)	0	0
New Jersey	5	0	0 (0–0.52)	0	0
New Mexico	176	2	0.01 (0–0.04)	0	0
Nevada	9	0	0 (0–0.34)	0	0
New York	28	1	0.04 (0–0.18)	0	0
Ohio	80	0	0 (0–0.05)	0	0
Oklahoma	677	30	0.04 (0.03–0.06)	0	0
Oregon	83	4	0.05 (0.01–0.12)	0	0
Pennsylvania	6	0	0 (0–0.46)	0	0
South Carolina	402	10	0.02 (0.01–0.05)	0	0
Tennessee	170	5	0.03 (0.01–0.07)	0	0
Texas	1285	53	0.04 (0.03–0.05)	0	0
Utah	5	0	0 (0–0.52)	0	0
Virginia	94	1	0.01 (0–0.06)	0	0
Wisconsin	4	0	0 (0–0.60)	0	0
West Virginia	47	0	0 (0–0.08)	0	0

*PEDV, porcine epidemic virus.

Technical Appendix Table 2. Feral swine samples sizes by yea, United States*

Year	No.	No. coronavirus-positive	Coronavirus seroprevalence (95% CL)	No. PEDV-positive	PEDV seroprevalence (95% CL)
2012	491	11	0.022 (0–0.037)	0	0
2013	2079	49	0.024 (0–0.011)	0	0
2014	2931	106	0.036 (0–0.002)	8	0.003 (0–0.005)
2015	2496	87	0.035 (0–0.011)	1	0 (0–0.002)

*PEDV, porcine epidemic virus.
